# European Academy of Cancer Sciences – position paper

**DOI:** 10.1002/1878-0261.12379

**Published:** 2018-09-21

**Authors:** Hans‐Olov Adami, Anton Berns, Julio E. Celis, Elisabeth de Vries, Alexander Eggermont, Adrian Harris, Harald zur Hausen, Pier Giuseppe Pelicci, Ulrik Ringborg

**Affiliations:** ^1^ Karolinska Institutet Stockholm Sweden; ^2^ Netherlands Cancer Institute Amsterdam The Netherlands; ^3^ Danish Cancer Society Research Centre Copenhagen Denmark; ^4^ University Medical Center Groningen – University of Groningen The Netherlands; ^5^ Gustave Roussy Cancer Campus Grand Paris Villejuif France; ^6^ University of Oxford UK; ^7^ German Cancer Research Centre Heidelberg Germany; ^8^ European Institute of Oncology IRSSC Milan Italy

**Keywords:** bridging research and healthcare, cancer research continuum, Comprehensive Cancer Centre, innovation of prevention, societal impact, therapy development

## Abstract

The European Academy of Cancer Sciences (EACS) is an independent advisory body of well‐recognised medical specialists and researchers striving to create a compelling interactive continuum of cancer research, from innovative basic research to implementation of state‐of‐the‐art evidence‐based cancer care and prevention. Achieving the above will entail bridging high‐quality basic and preclinical cancer research to research on prevention, early detection and therapeutics as well as improving coordination of translational research efforts across Europe. The latter is expected to be expedited through quality assuring translational cancer research in Comprehensive Cancer Centres – entities that link research with the healthcare system – and networks of cancer research centres. Achieving a critical mass of expertise, resources and patients is crucial. Improving late translational research, which involves clinical studies to assess effectiveness, and added value for the health care is also a high priority. Both high‐quality Big Data collections and the intelligent use of these data will promote innovation in cancer research and support outcomes research to assess clinical utility, quality of cancer care and long‐term follow‐up of treated patients. The EACS supports the mission‐oriented approach recently proposed by the European Commission in Horizon Europe to deal with major challenges and would like to persuade the EU and its member states to formally launch a mission in cancer to boost and streamline the cancer research continuum in Europe. Building a coherent translational cancer research continuum with a focus on patients and individuals at risk will require, however, foresight as well as the extensive and continuous provision of evidence‐based advice to inform policy.

**Abbreviations**
CCC: Comprehensive Cancer Centre
EACR: European Association for Cancer Research
EACS: European Academy of Cancer Sciences
EC: European Commission

## 1 The burden of cancer

Cancer is a major challenge for society, the healthcare systems and the growing number of affected patients. In the 40 European countries, cancer incidence in 2015 was 3.6 million with an expected increase to 4.3 million in 2035. The rise in the ageing population is a primary reason for the incidence trends, although cancer is also a significant cause of death before the age of 70. During the same 20‐year period, the annual number of patients dying from cancer in the European countries is predicted to increase from 1.8 to 2.3 million (Ferlay *et al*., 2013). In addition, the steady growth in the number of cancer survivors and the more chronic nature of the disease – with its own specific comorbidities and requirements for regular monitoring – will further increase the burden on the healthcare systems. In the European Union (EU), the cost of cancer was estimated at 126 billion euros in 2009 (Luengo‐Fernandez *et al*., 2013) with significant differences per capita between countries.

The continuously growing insights in cancer biology over the last decades have revealed an enormous complexity and heterogeneity of human malignancies. This complexity impacts the whole continuum of cancer research, which includes fundamental research, translational, prevention and clinical research, outcomes research, and cancer care. The combination of a rising incidence and prevalence, the increasingly chronic nature of the disease, combined with the complexity and associated costs of treatment – partly the result of the often‐exorbitant pricing of anticancer drugs – can easily lead to a collapse of the healthcare systems. As a result, substantial innovation in both prevention and treatment is urgently needed.

To deal with these challenges, Europe must take coordinated actions. Stakeholders to be involved include the EU commission, national governments, health organisations, professional societies, cancer centres, hospitals and research institutes. This position paper provides the viewpoints of the European Academy of Cancer Science (EACS; http://www.europeancanceracademy.eu) regarding the cancer research continuum and highlights specific areas where initiatives should be taken. The primary aim of the EACS is to increase awareness of these issues and to help developing action plans. By proposing effective incentives for collaboration between stakeholders in the different EU countries (and beyond) and by providing quality standards and assessment protocols for cancer centres and institutes, the EACS hopes – in collaboration with other organisations with overlapping aims – to contribute to an EU‐wide more effective treatment and prevention of cancer patients.

## 2 Present state of affairs

### 2.1 Addressing the fragmentation of cancer research

An extensive analysis of European cancer research was carried out in 2005–07 by the EU‐project ‘Eurocan+Plus’ initiated by former Commissioner Philippe Busquin and supported by the European Parliament. He considered that cancer research in Europe was fragmented and that many patients had no access to the diagnostic services and therapeutic treatments that they rightly demanded (http://www.europa.eu/rapid/press-release_SPEECH-02-408_en.htm): ‘There was a clear need to create a common European strategy for cancer research’. The cancer community was asked to improve coordination of cancer research activities in Europe through already existing funding mechanisms. One main recommendation of the project was the creation of a platform for translational cancer research, composed of interlinked centres with shared infrastructures and collaborative projects to facilitate rapid advances in knowledge and their translation into better cancer care and prevention. The ‘Eurocan+Plus’ highlighted the need for structuring translational cancer research, which by definition focusses on issues directly relevant for cancer patients. The aim was to identify strategies to promote innovation across the whole cancer research continuum (basic, prevention, preclinical, clinical and outcomes research).

The project also stressed the increased need for complex infrastructures to integrate the multiple components of the research continuum. The Comprehensive Cancer Centre (CCC), a structure that provides multidisciplinary academic expertise covering a substantial stretch linking research and health care, was deemed to be of critical importance. Due to a large number of cancer patient subgroups and increased demands regarding technological resources and competencies, a close collaboration between CCCs was considered essential; such partnership would provide the full spectrum of expertise and resources, as well as access to a sufficient number of patients, both prerequisites for innovative research and personalised/precision cancer medicine.

The concerns articulated in the ‘Eurocan+Plus’ project were taken seriously by the cancer community, which organised itself to provide evidence‐based advice to inform policy. The aim was to persuade the European Commission (EC) to support FP7 (Framework Programme 7) activities intended to structure translational cancer research and to improve cross‐border institutional collaborations. These efforts resulted in the funding of the EurocanPlatform project, which from 2011 to 2016 explored various strategies to organise translational research by bridging basic/preclinical research with clinical studies. One of the outcomes was the establishment of Cancer Core Europe, now a legal consortium consisting of seven large cancer centres (mainly CCCs) across Europe (Eggermont *et al*., 2014). Besides providing a model of how better coordination and critical mass might be organised, the bottom‐up initiative created an ecosystem where the three priorities of Commissioner Carlos Moedas, Open Science, Open Innovation and Open to the World could be interlinked, and where the social dimension of science becomes evident. However, this initiative also illustrated the many obstacles that can preclude effective cross‐border collaboration. In parallel, a consortium of nine cancer prevention centres was established (Cancer Prevention Europe) to support the complete cancer prevention research continuum. A Consortium Agreement was signed in February 2017, with present plans to incorporate two more centres.

Other deliverables of the project included the establishment of a designation methodology for CCCs of Excellence (for specific assessment of bench to bedside research), developed in collaboration with the EACS (Ringborg *et al*., 2018). Moreover, because educational activities with a focus on translational cancer research were limited, an annual international Summer School in Translational Cancer Research was established in 2012, with the intention of fostering collaboration between basic, translational and clinical researchers. The school is now sustainable under the umbrella of Cancer Core Europe.

The above developments emphasised the need for EU‐wide science policy as prerequisite for coordinating cutting‐edge cancer research, establishing adequate funding mechanisms, harmonising the regulatory environment and for eliminating disparities in cancer control among EU populations. Towards this end, the EACS was established in 2009 with support from ECCO (the European CanCer Organization). The EACS is now a legal structure whose primary goal is to boost innovation and research coordination, as well as to establish state‐of‐the‐art research infrastructures with outreach to local hospitals and healthcare organisations in order to achieve efficient and cost‐effective cancer prevention, treatment and care services throughout Europe.

### 2.2 Cancer prevention research

It is impossible to optimise cancer control by improving treatment only, and as a result, an integrated approach built on prevention, early detection and treatment is critical. An estimated 40% reduction of the cancer incidence can be achieved by fully implementing present knowledge of prevention strategies. Primary prevention aims at averting the exposure to carcinogens and other cancer‐promoting conditions, several of which are common to the main noncommunicable diseases. Here, proper legislation to encourage behavioural changes, vaccination technologies and medical prevention is a critical component. In particular, legislation focusing on reducing exposure to carcinogens, tobacco smoking being the most prominent example, is a key policy issue rather than a scientific one. Preventive cancer screening or secondary prevention, aimed at removing precursor lesions known with likelihood to progress to invasive cancer such as cervical cancer, colonic adenoma and melanoma, has shown to be effective. However, for other cancers, such as breast and prostate, the early lesions prone to progress to invasive cancers are more difficult to diagnose. Identifying premalignant lesions that are likely to progress is a strategic component of the research aimed at understanding the drivers of tumour initiation and tumour progression. For some common malignant diseases, screening technologies for early detection are in place, and population‐based screening programmes have been established in several European countries for cervical, breast and colorectal cancer. Critical research areas include the development of improved diagnostic technologies, as well as methodologies for follow‐up screening and outcome prediction. Algorithms that can predict outcome in individual patients combined with sophisticated devices to monitor clinically relevant parameters over time can revolutionise this field. Big Data approaches will likely play an important role in their development. A comprehensive manuscript describing the Cancer Prevention Europe strategy has recently been accepted for publication (Forman *et al*., 2018).

The level of financial support for research in prevention is roughly 7% of the cancer research budget. Optimal allocation of resources for the different research categories is a vital science policy question (Cancer Research Partnership).

### 2.3 Therapeutic research

Surgery and radiotherapy, increasingly combined with systemic treatment, can cure most patients with localised diseases. The great challenge is to make localised treatment less mutilating. Incremental advances are still made to make the treatment less harmful and to control advanced loco‐regional disease, but the great challenge is to control disseminated cancer using some form of systemic therapy. Therapeutic research focused on primary as well as disseminated cancer is receiving substantial emphasis thanks to the massive introduction of ‘omics’ techniques for the analysis of tumour samples and liquid biopsies. Fostered by cancer biology research, important progress has been made; sophisticated combination therapies and immunotherapies have been developed that appear to be effective in several cancer subtypes. However, the majority of disseminated cancers are resistant or develop resistance to systemic treatment.

Significant investments to understand the complexity of these tumours, their heterogeneity, their plasticity, their genetic and epigenetic drift, the role of the microenvironment and the failure of the immune system to recognise and eradicate these tumours require significant research efforts; basic cancer research has to provide the answers. Subsequently, the concepts coming from these more fundamental studies must be validated in relevant preclinical models before they are tested in clinical trials. For this latter translational part, well‐organised CCCs focused on designing innovative data‐rich and deep clinical trials and capable of swiftly recruiting a sufficient number of patients with a specific biomarker signature are critical. Here, EU‐wide collaboration of cancer research centres as exemplified by Cancer Core Europe is of pivotal importance.

The EACS believes that Cancer Core Europe provides a model for establishing similar consortia with sufficient critical mass (but not too large to keep the consortia manageable and flexible) to recruit sufficient numbers of patients. The advances made in trial designs, allowing patients to be treated according to a protocol based on molecular markers and response profiles are expected to create timely new insights that may lead to changes in clinical practice. These future trials will, however, also require advanced methodologic skills and challenging trade‐offs between statistical power and generalisability of findings from cancers with molecularly defined malignancies (Hunter, 2016).

### 2.4 Bridging research and health care

As pointed out above, insights gained in research can have a significant impact on the quality of cancer care; patients are not willing to wait for new diagnostic and treatment methodologies. To support innovation in cancer care, the CCC is a critical structure, since by definition a CCC integrates multidisciplinary cancer care with research and education. Prevention can be an integrated part of a CCC or a component of other infrastructures.

Although CCCs appear best equipped to catalyse this process, most clinical care is provided by clinics outside the CCCs. Consequently, CCCs must be charged with the responsibility to reach out and interact closely with these clinical centres concerning the quality of care, innovation and research collaboration. Europe has two accreditation bodies for CCCs, the OECI (Organization of European Cancer Institutes) and the German Cancer Aid. Moreover, the EACS offers a programme for Designation of CCCs of Excellence assessing the quality of translational cancer research. Therefore, it is important that accreditation of CCCs also assesses the geographical outreach as an independent parameter. Up‐to‐date clinical guidelines, based on clinical practice guidelines provided by professional organisations such as NCCN (National Comprehensive Cancer Network), ASCO (American Society of Clinical Oncology), ESMO (European Society for Medical Oncology), ESTRO (European Society for Radiotherapy and Oncology), ESSO (European Society of Surgical Oncology) and NICE (National Institute for Health and Care Excellence) covering the complete clinical pathway and linked to clinical cancer registries, are needed to guide the implementation and outcome of innovations within the healthcare systems. The capability to couple registries from different countries will be of critical importance to collect statistically significant data, given the increasing stratification of patients. By using real‐world data, the clinical utility of innovations can be evaluated, an important aspect being the long‐term follow‐up of cancer patients, which is presently an unmet need.

Cancer survivors represent an increasingly large population, and their well‐being depends on the outcome of therapeutic interventions, cancer care, post‐treatment monitoring, rehabilitation, tertiary prevention and, for some, palliative care. Currently, there is an impressive development of diagnostic technologies supporting both prevention and therapeutic disciplines. Supportive care, psychosocial oncology, rehabilitation and palliative care are increasingly important for a growing patient population living with cancer as a chronic disease. There is a need to structure research on health‐related quality‐of‐life issues, and hence, it is important that the accreditation methodologies in the long term will be able to assess all areas of cancer care and prevention.

Innovative academic cancer research has identified many new appealing drug targets. However, this has resulted in increased marketing of anticancer drugs and medical devices with insufficient information on effectiveness. An explosion of the cost of treatments due to the very high prices charged for drugs by many pharmaceutical companies is a threat to citizens’ access to state‐of‐the‐art therapies. This is an increasing concern because costs might deprive patients of effective treatment purely for economic reasons. This is not acceptable and requires recalibration of how inventions made by life sciences in academia – paid by society – can benefit patients maximally at an affordable cost.

At present, Cancer Core Europe and Cancer Prevention Europe are in the process of integrating therapeutics and prevention strategies to identify and remedy, in partnership, existing gaps in the cancer care continuum. By offering innovative approaches for cancer research, links to the healthcare systems, development of quality‐assured multidisciplinary cancer care and the assessment of long‐term outcomes, such distributed infrastructures are expected to serve as hubs to connect with other centres in Europe as well as in other continents.

Parallel to these developments, the EC recently decided to tackle global challenges by moving from a challenge‐driven to a mission‐oriented approach in Horizon Europe; missions open new possibilities to address significant societal challenges such as cancer, as for the first time there is an open discussion and analysis of the relationship between research and its social impact (http://europa.eu/rapid/press-release_IP-18-4041_en.htm). This development prompted Julio E. Celis and Dainius Pavalkis – members of the RISE High‐level Group advising Commissioner Carlos Moedas – to propose a mission in cancer (‘A mission‐oriented approach to cancer in Europe: a joint mission/vision 2030’) (Celis and Pavalkis, 2017) based on the activities and objectives of Cancer Core Europe and Cancer Prevention Europe. The mission states ‘by combining innovative prevention and treatment strategies in a sustainable state‐of‐the‐art virtual European cancer centre/infrastructure, it will be possible by 2030 to achieve long‐term survival of 3 out of 4 cancer patients in countries with well‐developed healthcare systems. Furthermore, the concerted actions will pave the way to handling the economic and social inequalities in countries with less‐developed systems’. The cancer mission targets the entire cancer research continuum, engages all the stakeholders and emphasises the social impact of cancer research. Moreover, it differs from the Moonshot programme in the United States, as it is a bottom‐up initiative from the cancer community that crystallised after many years of continuous work intended to provide evidence‐based advice to inform policy (Celis and Pavalkis, 2017).

No doubt, creating an effective cancer research continuum requires substantial resources. Therefore, the EACS wants, together with other EU cancer organisations, to persuade the EU and its member states to formally launch a mission in cancer to boost and streamline the cancer research continuum in Europe.

## 3 Vision statement

The EACS is an independent advisory body of well‐recognised medical specialists and researchers, placing science at the core of policies with the aim of reducing, at an affordable price, death and suffering caused by cancer in Europe.

## 4 Mission

### 4.1 The EACS strives to create a compelling interactive continuum of cancer research, from innovative basic research to implementation of state‐of‐the‐art evidence‐based cancer care and prevention at an affordable cost

A network of closely collaborating CCCs, each with outreach to local healthcare organisations, will be needed to create a system in which access to first‐rate prevention, diagnosis, treatment, cancer care, post‐treatment surveillance and palliation can be guaranteed at an affordable cost. To secure quality and foster effective interactions requires legislation and incentives from governmental organisations.

The EACS wants to specifically emphasise the issues that will require further attention to achieve the above.

#### 4.1.1 Support for high‐quality basic and preclinical cancer research

Many of the advances made in recent decades have been catalysed by basic research, often conducted outside the cancer research arena. Oncogenes were first recognised by virologists, and virus research has contributed significantly to our understanding of what causes cells to become malignant. Understanding the principles of genetics, genome organisation and how mutations are acquired and repaired or not have been of fundamental importance, as are the insights gained in cell biology and development, and the many circuits in cells that control cell behaviour. Much of the progress was made possible through technological developments; the sequencing of genomes, RNA and proteins; the capacity to manipulate genomes of cells; and model organisms have been invaluable. Single cell analyses provide further insight into the complexity of tumours and their microenvironments. The knowledge gathered on the way has made it possible to mobilise the immune system of cancer patients to reject their own tumour.

The Next Generation Sequencing technology in conjunction with high capacity computing (and the promise of Artificial Intelligence) will permit the integrative analysis of large datasets. Datasets from cancer patients have become appealing for formulating and validating basic research questions relevant for effective prevention, early detection and therapeutic intervention. Over and over again, nature appears much more complex than we thought and we continue to be astonished by the molecular strategies that evolved during evolution and that are hijacked by cancer cells. Understanding how nature works should remain an essential component of cancer research, as it often permits unexpected leaps rather than small steps forward.

The EACS is closely monitoring these developments. Presently, there is a Memorandum of Understanding between the EACS and the European Association for Cancer Research (EACR) to collaborate regarding strategies to cover the continuum from basic to clinical research and its subsequent implementation.

#### 4.1.2 Creation of collaborative networks focussed on primary prevention

Understanding of the aetiology of cancer requires high‐quality epidemiological research with ample access to registries and patient databases. This could lead to the identification of risk factors and optimally to their removal from the environment. In addition, however, it is equally important to link this to molecular biological data, to genetics and to available immunological techniques. Research focusing on active prevention measures, which includes vaccination (primary prevention), secondary prevention by early diagnosis and removal of precursor lesions, early removal of existing tumours by surgery, radiation, chemical or immunological interventions should be promoted. This can substantially reduce the cancer burden and thereby considerably reduce the demands on the healthcare system. In particular, this might contribute to a reduction of the relatively high burden of cancer in the socioeconomically less‐privileged individuals in our society.

#### 4.1.3 Support for research in the area of early detection

Early detection is widely seen as an effective strategy to reduce the risk of dying from cancer. Whereas screening has shown value in a subset of cancer types (cervix, colon, melanoma), in many cases it remains difficult to detect cancer early enough to eliminate the disease by local treatment only. Furthermore, even when lesions can be detected early, it remains questionable what action is most appropriate. We still lack informative biomarkers to identify lesions with high risk of progressing to lethal disease, as it is the case for breast and prostate. To detect such lesions with sufficient specificity and sensitivity is still a major bottleneck. The EACS strongly encourages research within this area.

#### 4.1.4 Support for translational research aimed at identifying new innovative therapeutic interventions

In spite of the generation of many highly specific drugs against oncogenic drivers, their therapeutic effects are in general quite modest. This is almost invariably due to intrinsic or acquired resistance. Therefore, there is a need for more sophisticated intervention strategies, based on detailed insight into the wiring circuits and heterogeneity of individual tumours. Specific drug combinations and drug scheduling strategies need to be explored to overcome this resistance. Predictive cancer medicine is considered important, including prediction of both antitumour and side effects. This also applies to immunotherapy. Finding better predictors of response, next to more effective mobilisation of the immune system while limiting the side effects, as well as strategies to prevent or overcome escapes demand substantial investments.

This segment of early translational research already received significant support, but raising funds to test some of the promising findings in small, well‐stratified patient populations is an increasing bottleneck. It is essential that phase I/II clinical trials focus on innovative concepts with ample data collection, rather than iteration of research projects in which the same intervention concept is explored in many parallel trials (e.g. the many PD1/PDL1 antibody trials). It is important to note that predictive cancer medicine is important for guiding both systemic treatment and complementing applications of localised treatments, such as radiation therapy and surgery.

#### 4.1.5 Quality assurance of research environments and development of strategies to improve the infrastructures

Translational research bridges basic cancer research with clinical research. It requires an environment of a CCC in which basic cancer researchers closely interact with clinical colleagues in a mutually stimulating academic environment. The effectiveness of such an entity is determined by the quality of the research, and by how basic and clinical researchers interact and engage in joint projects to test new concepts. Such collaboration might originate from basic research or be inspired by insights obtained from the analysis of clinical data. The EACS has developed a protocol to assess the quality of a CCC and certify such centres as a Centre of Excellence if they meet defined requirements. It permits institutions to further improve their overall quality as a CCC for the benefit of patients. The EACS will approach EU and government organisations, as well as charities, to put incentives in place for Institutes to join this programme.

The EACS also wants to develop quality assurance programmes for academic entities that are not part of a CCC, but significantly contribute to distinct segments in the cancer research trajectory, for example cancer biology, genetics, systems biology, drug development, local precision treatment, epidemiology, outcomes research, socio‐economics, health‐related quality‐of‐life research, medical prevention and palliative care.

#### 4.1.6 Establishment of collaborative networks with aligned diagnostic and treatment services

Early translational cancer research is of vital importance. Sufficient capacity to swiftly test new promising concepts in stratified patient populations within data‐rich innovative clinical trials should have priority. This is a major challenge. Cancer Core Europe and Cancer Prevention Europe are exploring how this can be best implemented in more massive, collaborative structures, and as such, these organisations can serve as a template for the creation of similar parallel structures. To build expertise, these collaborative networks should have access to funding mechanisms that facilitate the creation of infrastructures to effectively conduct such trials. Substantial funding of these activities (including the phase I/II trials) through governmental organisations or health insurers (with umbrella arrangements with pharma) would help to improve the quality, limit iterative research, accelerate trial execution and result in more effective treatments at an affordable cost.

#### 4.1.7 Support for research that can assess the effectiveness of prevention, early detection and clinical interventions

We need to evaluate whether new treatment protocols based on clinical trials retain the claimed benefits of broader implementation and assess the effectiveness for patients and the consequences for the healthcare system. The latter is particularly important for new anticancer agents that go to market without sufficient information about clinical effectiveness related to survival and health‐related quality of life, as well as consequences for the healthcare system. Health economics is a missing piece and should become a structural component of the cancer research continuum. Agreement on criteria for evidence and definition of clinical effectiveness is important in the context of personalised/precision cancer medicine. Reproduction of research outcomes in the healthcare systems by real‐world data from quality‐assured clinical registries is a central component in the assessment of the clinical utility of innovation and quality of care. Health‐related quality‐of‐life research should have a higher priority, being an essential outcome component of therapeutic development and should include analyses of long‐term surviving patients.

The EACS supports the integration of prevention and therapeutics research as executed by Cancer Core Europe and Cancer Prevention Europe. Obviously, early detection is relevant both for secondary prevention and therapeutic intervention, since cancer at an early phase of development can be more effectively treated, reducing mortality and morbidity. The EACS will continue to identify and work towards bridging any gaps in the cancer research continuum from basic research to the assessment of outcomes for cancer survivors. The multiple aspects of outcomes research are at present not well coordinated and structured, and the EACS will work for a more consistent outcomes research endeavour.

#### 4.1.8 Development of open science, open innovation and open to the world

Europe has achieved some critical goals in the last decades with the creation of large consortia for cancer therapeutics and prevention research. The possibility to rollout a European infrastructure for advanced translational cancer research has now come within reach, since accreditation methodologies for CCCs and a programme for assessment of translational cancer research and designation of CCCs of Excellence are now operational. With high‐quality collaboration between quality‐assured cancer research centres, there is a possibility in the long‐term to establish a virtual European Cancer Institute, as requested by the former Commissioner Philippe Busquin and suggested by the ‘Eurocan+Plus’ project.

#### 4.1.9 Adoption of innovations by healthcare organisations

Innovations with rigorously proven benefits for patients must be swiftly included in clinical guidelines to promote broader implementation. Healthcare organisations and CCCs need to facilitate this process, which includes implementation of prevention and early detection programmes. New applications taking advantage of the increasing sophistication of recognition software, data integration and new algorithms (with upcoming Artificial Intelligence) offer exciting new perspectives. Traditionally, health organisations are somewhat conservative and not in the best position to stimulate such developments. Here, the EU could take initiative to install a small board of experts to catalyse testing and implementation of promising new concepts. The EACS should aspire to take the lead in establishing such a board.

#### 4.1.10 Support for high‐quality Big Data collections and intelligent use of these data

There is little doubt that interrogation of large data sets can yield unexpected new insights, which will benefit patients (e.g. more effective prevention and treatment). In the past, many datasets appeared incomplete or of insufficient quality to make them relatively worthless: ‘garbage in, garbage out’. Whereas collecting high‐quality molecular data (next generation sequencing of genomes, epigenomes and transcriptomes) is becoming routine, this is still less the case for the associated clinical data. Establishing extensive quality‐controlled patient records that can be shared between institutions within Europe is even more of a challenge, also for formally collaborating institutes such as those constituting Cancer Core Europe. Thus, it is urgent to reach an agreement about the critical set of data (and their quality parameters) that needs to be collected, to make them useful for big data analysis. The algorithms to mine the data optimally, including Artificial Intelligence strategies, will be needed as well, but are unlikely to constitute a bottleneck. The EACS will pay specific attention to these quality aspects when advising and assessing institutions in their designation programme.

#### 4.1.11 Development of initiatives in the area of digital health

A specific combination of easily measurable parameters might turn out to be indicative for early disease. Furthermore, simple measurements might also permit monitoring of disease progression or relapse early on. Digital health approaches could play a prominent role in this. The EACS wants to promote these developments by inviting experts in digital health to join forces with renowned cancer pathologist to explore this specific field and to report on it.

## 5 European Cancer Science Policy: an unmet need

To address the issues pinpointed above will require foresight as well as the extensive and continuous provision of evidence‐based advice to inform policy. Towards this end, the EACS has strengthened the policy work by establishing a Scientific Policy Committee based on multiple competencies. Policy issues under consideration include: (a) the organization of research and funding mechanisms needed to develop sustainable translational cancer medicine; (b) governance and expansion of existing consortia for therapeutics and prevention; (c) structuring the interaction between basic/preclinical research centres, professional clinical and prevention research organisations, and consortia of CCCs; (d) updates of accreditation methodologies for CCCs as well as Designation of CCCs of Excellence; (e) structuring of the health care by expanding the number of CCCs with formalised responsibility for outreach areas to bridge research and multidisciplinary health care and decrease inequalities in the European countries; (f) development of strategies to achieve cost‐effective cancer care and prevention in the long run; (g) long‐term follow‐up for assessment of cancer survivorship; (h) the engagement of all the key stakeholders, including patient organisations; and (i) sustainability of actions.

The Committee will prioritise areas of policy action taking into consideration the urgent need to link research with the healthcare systems as well as the opportunities that a cancer mission will bring to ensure that in the long run, science‐driven and social innovations reach patients across the healthcare systems in Europe.

## Author contributions

All authors contributed to the writing of this article.
